# The impact of passive alveolar molding vs. nasoalveolar molding on cleft width and other parameters of maxillary growth in unilateral cleft lip palate

**DOI:** 10.1007/s00784-023-05119-7

**Published:** 2023-06-23

**Authors:** Robert Parhofer, Andrea Rau, Karin Strobel, Lina Gölz, Renée Stark, Lucas M. Ritschl, Klaus-Dietrich Wolff, Marco R. Kesting, Florian D. Grill, Corinna L. Seidel

**Affiliations:** 1 Department of Oral and Maxillofacial Surgery, Friedrich-Alexander-Universität (FAU) Erlangen-Nürnberg, Gückstr. 11, 91054 Erlangen, Germany; 2 Department of Oral and Maxillofacial Surgery/Plastic Operations, Universität Greifswald, Fleischmannstr. 8, 17489 Greifswald, Germany; 3 Department of Orthodontics and Orofacial Orthopedics, Friedrich-Alexander-Universität (FAU) Erlangen-Nürnberg, Gückstr. 11, 91054 Erlangen, Germany; 4 Institute for Health Economics and Healthcare Management, Helmholtz Zentrum Munich, Neuherberg, Ingolstädter Landstr. 1, 85764 Oberschleißheim, Germany; 5 Department of Oral and Maxillofacial Surgery, Technische Universität München, Lagerstr. 3, 81675 Munich, Germany; 6 Department of Orthodontics and Orofacial Orthopedics, Ludwig-Maximilian-Universität (LMU) München, Goethestr. 70, 80336 Munich, Germany

**Keywords:** Nasoalveolar molding, Passive alveolar molding, Cleft lip palate, Presurgical orthodontics, Maxillary growth

## Abstract

**Objective:**

Passive alveolar molding (PAM) and nasoalveolar molding (NAM) are established presurgical infant orthodontic (PSIO) therapies for cleft lip palate (CLP) patients. PAM guides maxillary growth with a modified Hotz appliance, while NAM also uses extraoral taping and includes nasal stents. The effects of these techniques on alveolar arch growth have rarely been compared.

**Material and methods:**

We retrospectively compared 3D-scanned maxillary models obtained before and after PSIO from infants with unilateral, non-syndromic CLP treated with PAM (*n* = 16) versus NAM (*n* = 13). Nine anatomical points were set digitally by four raters and transversal/sagittal distances and rotations of the maxilla were measured.

**Results:**

Both appliances reduced the anterior cleft, but NAM percentage wise more. NAM decreased the anterior and medial transversal width compared to PAM, which led to no change. With both appliances, the posterior width increased. The alveolar arch length of the great and small segments and the sagittal length of the maxilla increased with PAM but only partially with NAM. However, NAM induced a significant greater medial rotation of the larger and smaller segment compared to PAM with respect to the lateral angle.

**Conclusions:**

NAM and PAM presented some significant differences regarding maxillary growth. While NAM reduced the anterior cleft and effectively rotated the segments medially, PAM allowed more transversal and sagittal growth.

**Clinical relevance:**

The results of this study should be taken into consideration when to decide whether to use PAM or NAM, since they show a different outcome within the first few months. Further studies are necessary regarding long-term differences.

**Supplementary Information:**

The online version contains supplementary material available at 10.1007/s00784-023-05119-7.

## Introduction

Cleft lip and palate (CLP) are one of the most common congenital malformations with an incidence of 1:600 worldwide [1]. The extent of CLP can vary from a small lip cleft to a complete cleft affecting the lip, alveolar crest and palate and can occur bilaterally or unilaterally (uCLP) [2]. Nowadays, prenatal detection of CLP is possible between the 14th and 35th week of pregnancy using sonographic examination [3]. Especially in severe cleft types, treatment can be challenging and long-lasting during childhood and adolescence, sometimes even until adulthood. Hence, patients with CLP malformation require interdisciplinary treatment strategies by specialists, e.g., orthodontists, oral and maxillofacial surgeons, paediatricians, otorhinolaryngologists, speech therapists and dentists [2, 4]. While there is no standardized national or international therapy concept and definitions differ, overall CLP treatment can be divided into primary and secondary treatment. Primary treatment covers presurgical infant orthopaedic (PSIO) therapy as well as the surgical procedures of lip and palate reconstruction [2, 4]. Secondary treatment refers to functional or aesthetic improvements after primary cleft closure, e.g., presurgical orthodontic treatment prior to surgical secondary alveolar bone grafting.

PSIO is often necessary within the first days after birth lasting until lip and palate closure at 4–6 and 10–12 months of life, respectively [2, 5]. Shortly after birth, the most important function of PSIO is the separation of the nasal and oral cavity to improve feeding and prevent the tongue from expanding the palate cleft further. Another treatment goal is to reduce the extent of the cleft and navigate the growth of the alveolar segments. This navigation can be performed using active or more passive methods [2, 4]. An example for an active or even invasive PSIO is the Latham appliance, in which pins are placed in the alveolar bone. With these pins, the segments can then be actively drawn towards each other [6, 7]. However, the use of invasive PSIO techniques like Latham has decreased since studies showed they resulted in a higher frequency of anterior and buccal cross-bite and anterior open bites [8, 9].

Two more passive and well-known PSIO techniques are passive alveolar molding (PAM), which uses a modified Hotz appliance [10, 11], and nasoalveolar molding (NAM) according to Grayson [4, 12–16]. The principle of both techniques is to guide alveolar arch growth using functional appliances (acrylic plates). This can be achieved by grinding out the plate in the area of the alveolus as well as by adding acryl to certain areas of the plate. Treatment with PAM solely uses passive growth guidance by directing the movement of the alveolar segments using these grinding strategies [10, 11]. On the other hand, NAM not only uses the grinding and adding strategy but also has a more active effect on the alveolus by using extraoral taping on the cheeks, thus applying direct transversal forces on the lip segments and indirectly exerting pressure on the alveolar ridge [4, 12–14]. Additionally, NAM also uses one extraoral nasal stent for uCLP malformation, which helps to form the flattened and asymmetrical nostrils prior to lip surgery. In bilateral CLP cases, an additional extraoral stent is used to extend the columella [4, 10, 14–18]. Overall, nasal cartilage is shaped best after birth due to high oestrogen levels [19].

The effects of PAM and NAM treatment approaches have been described and analysed individually in some studies and the effectiveness of the nasal stent in NAM has also been investigated in other studies [20–25]. However, so far, the possible differences between the two techniques in relation to the growth and change of the alveolar arch of the maxilla have not been evaluated systematically in a comparative study design. Knowledge about the change in cleft width, the transversal and sagittal growth and the rotation of the maxillary segments is of high value for orthodontists and cleft surgeons alike. Hence, the aim of our study was to evaluate the effects of PAM and NAM on alveolar arch parameters in infants with unilateral, non-syndromic CLP, using 3D-scanned maxillary models obtained in the first week of life and after completion of the presurgical orthopaedics therapy before lip surgery.

## Patients and methods

For this purpose, two patient cohorts from two German university cleft centres were compared in a retrospective study. The PAM technique was being used exclusively at the University Hospital Erlangen and the NAM technique was being used exclusively at the Klinikum rechts der Isar of the Technical University of Munich. To be included in the study, patients had to meet all of the following criteria below.

1. Complete unilateral cleft lip palate
2. No association to a syndrome
3. Date of birth between 01.01.2010 and 31.12.2017
4. Two high quality plaster models needed to be available, one of the first week of life and one from the 10th to 20th week of life (after PSIO, before lip surgery)
5. Treatment with PSIO, either with PAM at the University Hospital Erlangen or with NAM at the Klinikum rechts der Isar of the Technical University of Munich
6. Caucasian ethnicity

Patient selection was not performed by the doctors that performed the treatment to ensure that no bias would result. Patient selection was performed according to patients lists and the presented inclusion criteria were investigated by one of the authors, who was not involved in patient treatment.

### PAM cohort

The patient cohort for the PAM therapy originated from the Dental Orthodontics Clinic of the University Hospital Erlangen of the Friedrich-Alexander University Erlangen-Nuremberg. All patients treated for CLP between 2010 and 2017 were inspected (*n* = 289). Patients without a complete CLP were excluded, leaving 64 patients. Of these, only 33 patients had two plaster models, which were usable for the second treatment time point. Another 17 patients were excluded because they did not have a uCLP or had a syndromale association of their uCLP, resulting in a total number of 16 patients with PAM therapy. All patients fulfilling inclusion criteria with complete, non-syndromic uCLP born between 2010 and 2017 were selected (*n* = 16). The cohort consists of 7 female and 9 male patients. Detailed data about the patients’ characteristics is presented in supplementary Table 1. Ethical approval was granted from the Ethics Committee of the University of Erlangen (3_20Bc).

### NAM cohort

The NAM patient cohort originated from the Clinic for Oral and Maxillofacial Surgery at the Klinikum rechts der Isar of the Technical University of Munich. For the selection, a list of patients which were treated with NAM between 2009 and 2018 was available (*n* = 97). Nine patients were excluded because they were born before 2010 or after 2017. Another three patients were excluded because of syndromale association, leaving 85 patients. Further exclusions occurred because of bilateral CLP (*n* = 34) and incomplete uCLP (*n* = 17) leaving only 34 patients. Of these, 21 patients did not have plaster models, which were usable for the second treatment time point. Thus, 13 patients with complete, non-syndromic, uCLP born between 2010 and 2017 were included in our evaluation of NAM. This cohort consisted of one female and 12 male patients. Detailed data about the patients’ characteristics can be found in supplementary Table 2. Ethical approval for a retrospective analysis of CLP cases was granted from the Ethics Committee of the Technical University of Munich (2022-378-S-SR).

### Material

Only plaster models, which met certain quality criteria, were selected for 3D scanning, e.g., models with major defects in the area of the alveolar bone and palate were excluded. The plaster models were scanned 3-dimensionally at the University B (D501 scanner from 3Shape Denmark; precision: 25 µm according to manufacturer). The scanned plaster models were saved as STL files and imported into the software GOM-Inspect (GOM-Inspect 2020), which can be used for dentistry 3D techniques [26–28]. Afterwards, four raters, two orthodontists specialized in cleft treatment and two oral maxillofacial surgeons, set the anatomical points on the 3D-scanned digital models (each rater set all points).

### Measuring points

The anatomical points were selected on the basis of previously published studies using plaster models with CLP [20, 21, 29, 30] and defined points are described in Table 1. However, only anatomical points were used, since we found large deviations for constructed points in an inter-rater analysis prior to the beginning of the study. All four raters set anatomical and constructed points on eight 3D models. While the constructed points had standard deviations of 1.9 to 2.1 mm, the anatomical points only had standard deviations of up to 1.1 mm. The only constructed point, that was used, is the centre point between the two tuber points, which was not placed by raters but calculated after the placement of both tuber points by raters. Each rater set the anatomical points on the digital models with the software GOM-Inspect. In the end, four sets of coordinates were available for each anatomical point. The coordinates were then exported into an Excel file (Microsoft Office 365). The point coordinate with the greatest deviation was excluded (basically a best-three-out-of-four principle). Based on the other three points, the centre point was mathematically calculated.

**Table 1:** Anatomical and mathematically calculated points set on the maxilla by four raters

Abbreviation	Measuring points	Definition
Ps/Pg	Pol points *Anatomical point*	Anterior most mesial alveolar ridge point on the small and large segment, respectively; the shortest connecting line in the anterior cleft region lies between the two points
C1s/C1g	Mesial Caninus points *Anatomical point*	Intersection of the lateral sulcus (mesial of the canine germ) and the highest point of the alveolar ridge Highest point mesial of the lateral sulcus
C2s/C2g	Distal Caninus points *Anatomical point*	Intersection of the lateral sulcus (distal to the canine germ) and the highest point of the alveolar ridge Highest distal point of the lateral sulcus
Tks/Tkg	Tuber points *Anatomical point*	Tuber points, most distal points of the alveolar ridge
IP	Interincisal point *Anatomical point*	Intersection of the alveolar ridge line with the line connecting the incisive papilla and the tectolabial frenulum
MT	Centre point of the tuberial line *Constructed point*	Centre point between the tuber points (mathematically constructed by Excel)

### Distances measured

**Fig. 1:**
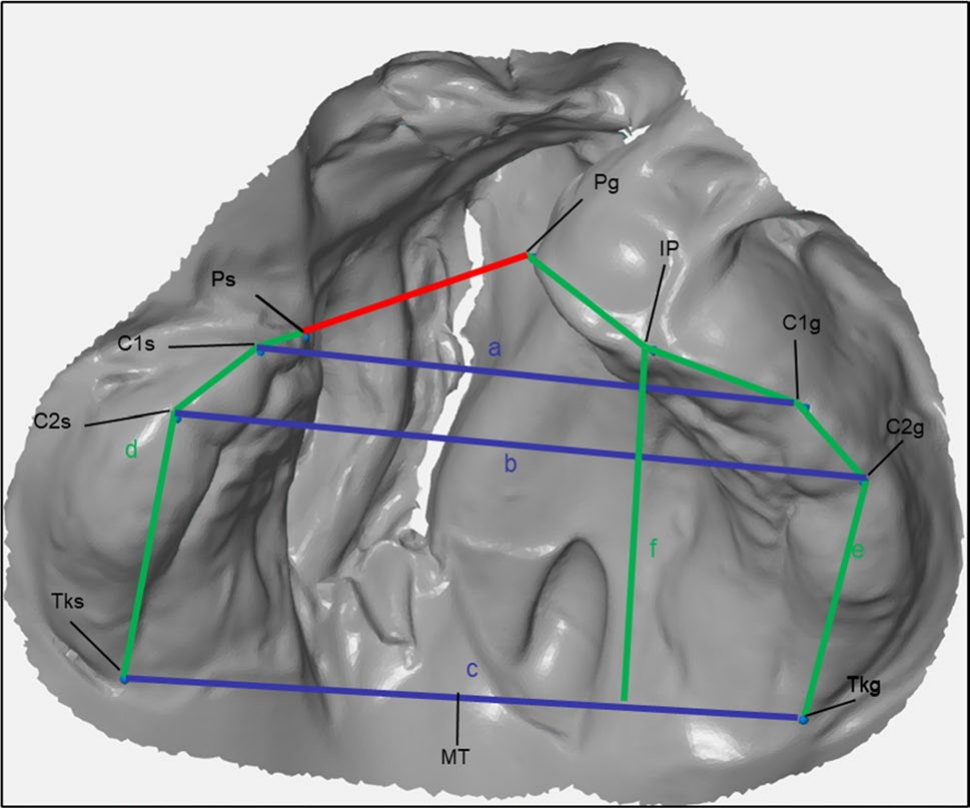
Points set and measured distances on example model in GOM-Inspect; red: anterior cleft; blue: anterior (**a**), medial (**b**), posterior (**c**) maxillary width; green: small (**d**) and great (**e**) alveolar arch length, sagittal maxillary length (**f**)

**Fig. 2:**
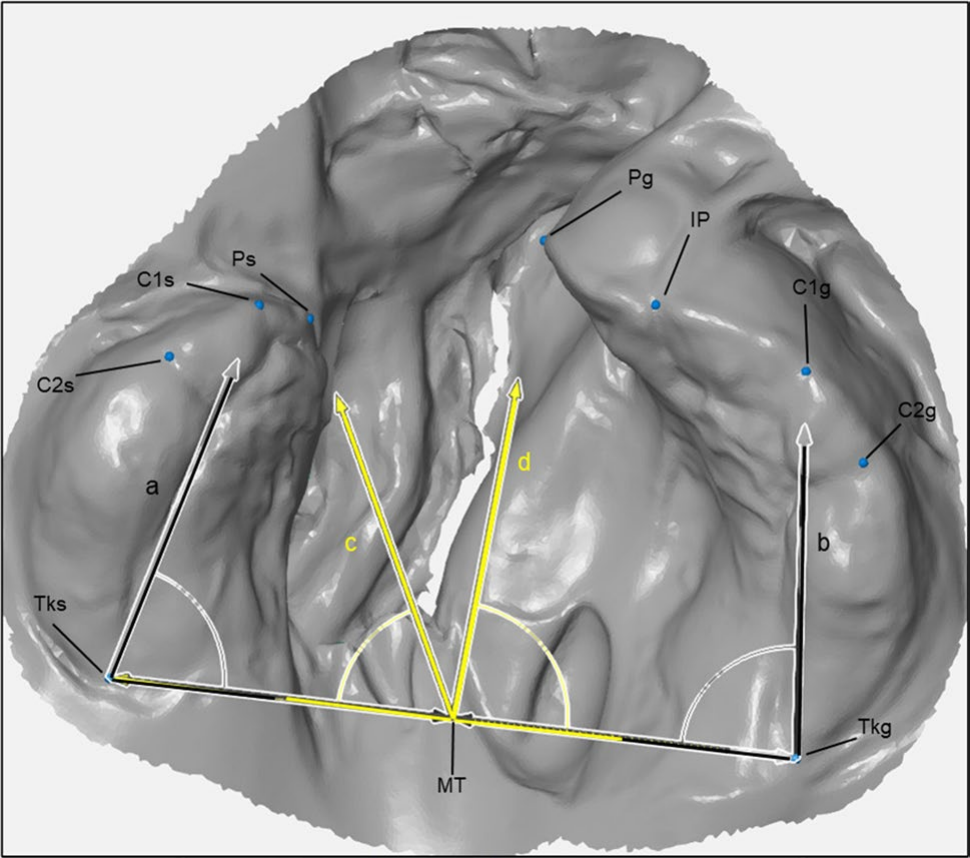
Measured angles on example model in GOM-Inspect; black: lateral angle of small (**a**) and great (**b**) segment; yellow: medial angle of small (**c**) and great (**d**) segment

Using the centre points, various distances and angles were calculated mathematically using Excel. Four transversal distances and three sagittal distances were measured. The measured distances are shown in Fig. 1, and the four angles are shown in Fig. 2. An overview of the measured distances and angles is shown in Table 2.

**Table 2:** Measured distances and angles on the maxilla

	Measuring points
Transversal distances	
Anterior cleft	Pg-Ps
Anterior maxillary width	C1g-C1s
Medial maxillary width	C2g-C2s
Posterior maxillary width	Tkg-Tks
Sagittal distances	
Alveolar arch length great segment	Pg-Tkg = Pg-IP + IP-C1g + C1g-C2g + C2g-Tkg
Alveolar arch length small segment	Ps-Tks = Ps-C1s + C1s-C2s + C2s-Tks
Sagittal maxillary length	IP-Tkg-Tks
Rotations	
Lateral angle of the great segment	C1g-Tkg-Tks
Lateral angle of the small segment	C1s-Tks-Tkg
Medial angle of the great segment	Pg-MT-Tkg
Medial angle of the small segment	Ps-MT-Tks

### Statistics

Both groups were initially examined if there was a statistical difference between the distances that were measured, and to evaluate if the distances are comparable, for this, a *t*-test was performed. There was no statistical difference in the distances that were measured (supplementary Table 3). Then both cohorts were examined with a two-sided *t*-test, as to whether changes in the calculated data were significantly different from zero. The two techniques were initially compared with a *t*-test and then using a mixed model-regression analysis, which adjusted for time interval of the two models and time of the treatment. The effect size was calculated with the criteria of Cohen’s *d*. All statistical analyses were performed with SAS (SAS 9.4) and a *p*-value of < 0.05 was considered statistically significant.

## Results

**Fig. 3:**
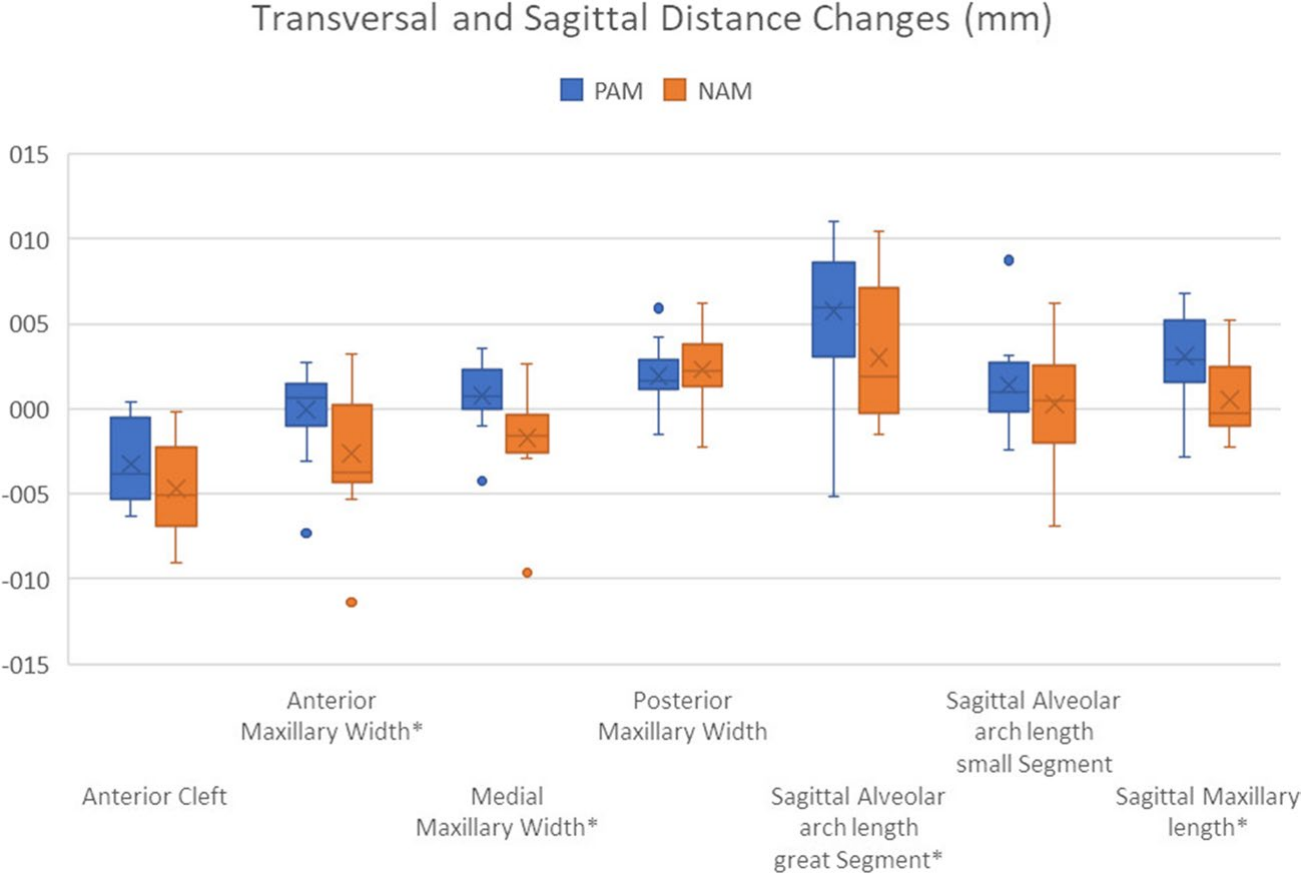
Comparison between changes of transversal and sagittal distances; *significant difference between PAM and NAM

**Fig. 4:**
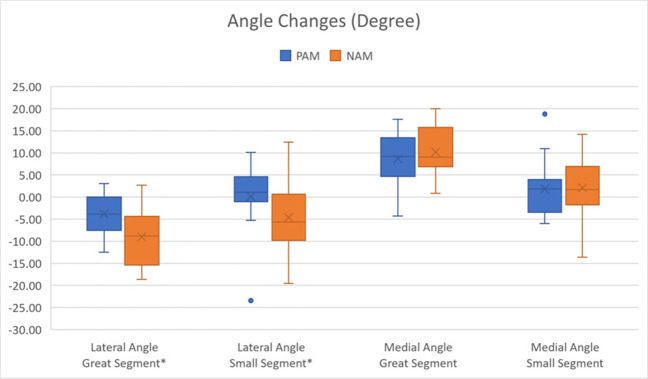
Comparison between changes of angles; *significant difference between PAM and NAM

Table 3 shows the mean changes of PAM and NAM and compares the pre- and post-treatment results for each technique as well as comparing the two techniques. Figures 3 and 4 illustrate the variance of the changes. Both appliances reduced the anterior cleft width significantly (PAM: − 3.25 mm; NAM: − 4.70 mm; *p* ≤ 0.0001). If the change is considered in mm, NAM reduced the cleft more than PAM, but the difference was not significant (*p* = 0.0968). However, if the percentage change is considered, there was a significant difference (*p* = 0.0227; NAM: − 50.35%, PAM: − 32.17%). This also revealed in the effect size which was 0.5750 calculated for change in whole numbers, while it was 0.8267 calculated for percentage change.

**Table 3:** Changes of distances/angles with PAM and NAM; *p*-value change to zero: significance of the change in the distance compared to no change; comparison of change *p*-value: significance of the difference between the change in PAM and NAM; bold: significant *p*-values (*p* < 0.05)

	Mean change in PAM	Change to zero in PAM *p*-value	Mean change in NAM	Change to zero in NAM *p*-value	Comparison in change *p*-value
Anterior cleft (mm)	− 3.25 ± 2.30	** < 0.0001**	− 4.70 ± 2.77	** < 0.0001**	0.0968*
Anterior maxillary width (mm)	− 0.07 ± 2.47	0.9160	− 2.60 ± 3.90	**0.0330**	**0.0038**
Medial maxillary width (mm)	0.79 ± 1.88	0.1152	− 1.72 ± 2.93	0.0557	**0.0003**
Posterior maxillary width (mm)	1.96 ± 1.69	**0.0003**	2.31 ± 2.14	**0.0022**	0.8351
Alveolar arch length great segment (mm)	5.77 ± 4.01	** < 0.0001**	3.03 ± 4.15	**0.0218**	**0.0288**
Alveolar arch length small segment (mm)	1.39 ± 2.50	**0.0422**	0.28 ± 3.69	0.7863	0.2736
Sagittal maxillary length (mm)	3.12 ± 2.35	** < 0.0001**	0.55 ± 2.41	0.4297	**0.0030**
Lateral angle great segment (°)	− 3.78 ± 4.26	**0.0029**	− 9.01 ± 6.63	**0.0004**	**0.0089**
Lateral angle small segment (°)	0.17 ± 7.29	0.9281	− 4.62 ± 8.93	0.0864	**0.0319**
Medial angle great segment (°)	8.67 ± 5.82	** < 0.0001**	10.20 ± 6.13	** < 0.0001**	0.6606
Medial angle small segment (°)	1.84 ± 6.37	0.2667	2.10 ± 7.59	0.3388	0.6719

As for the maxillary transversal dimensions (Fig. 3), there was a significant difference in the change between the two appliances in the anterior and medial maxillary width (anterior: *p* = 0.0038, medial: *p* = 0.0003). While with PAM, the anterior and medial maxillary width did not change significantly (− 0.07 mm, + 0.79 mm, respectively), NAM reduced both distances but only the anterior significantly (anterior: − 2.60 mm, *p* = 0.0330; medial: − 1.72 mm, *p* > 0.05). With both appliances, a significant growth was seen in the posterior maxillary width (NAM: + 2.31 mm, *p* = 0.0022; PAM: + 1.96 mm, *p* = 0.0003), and thus there was no significant difference between NAM and PAM (*p* = 0.8351).

Regarding the sagittal distances, significant differences between NAM and PAM were also detectable (Fig. 3). With PAM, the sagittal alveolar arch length of the great segment (*p* = 0.0288) and the sagittal length of the maxilla (*p* = 0.0030) increased significantly compared to NAM. There was significant growth of the sagittal alveolar arch length of the great segment with both appliances, however, with PAM (+ 5.77 mm, *p* ≤ 0.0001) more than with NAM (+ 3.03 mm, *p* = 0.0218). While the sagittal length of the maxilla significantly extended using PAM (+ 3.12 mm, *p* ≤ 0.0001), the change in length was not significant using NAM (+ 0.55 mm). Further, with PAM, the sagittal alveolar arch length of the small segment increased significantly (+ 1.39 mm, *p* = 0.0422), while there was no significant elongation using NAM (+ 0.28 mm). However, the difference was not significant between NAM and PAM (*p* = 0.2736).

Concerning rotation of the segments (Fig. 4), we measured a significant difference between NAM and PAM regarding the lateral angles, with greater mesial rotation using NAM (*p* < 0.04). While the great segment was significantly rotated medially with both appliances (lateral measurements: NAM: − 9.01°, *p* = 0.0004; PAM: − 3.78°, *p* = 0.0029; medial measurements: NAM: + 10.20°, *p* < 0.0001; PAM: + 8.67°, *p* ≤ 0.0001), the small segment was rotated medially with NAM (− 4.62°) and laterally with PAM (+ 0.17°) but the rotation for both was not significant.

## Discussion

The results showed that there are some significant differences between PAM and NAM concerning the growth and change in maxillary alveolar arch pattens. While for both techniques a reduction of the anterior cleft width was found, it was more pronounced with NAM. NAM also reduced the anterior and medial width of the maxilla, while the posterior width increased in both groups. In contrast, with PAM, the anterior and median transverse width was stabilized and all sagittal parameters showed significant growth. Both segments rotated more medially using NAM than using PAM considering lateral angle measurements, while PAM reduced the collapse of the small segment to the medial.

Summarizing the results, it is noticeable that some distances decreased with NAM, which did not change with PAM such as the anterior and medial maxillary width. This relates to the fact that with NAM, transversal forces are applied using extraoral tape leading to a significantly greater medial rotation of both segments and thereby reducing these two distances. The enhanced medial rotation can also explain the smaller increase in the sagittal length of the maxilla compared to PAM as well as the more significantly reduced anterior cleft width. Looking at the same distances in PAM, we found that the transversal anterior and median distances did not show any change. Moreover, with PAM treatment the alveolar arch length of the great segment as well as of the sagittal maxillary length increased significantly more. This can be explained by the use of more passive forces promoting more transversal and sagittal growth. Moreover, the greater segments did not show such a pronounced medial rotation and the smaller segment rotated laterally with PAM. The reduction of these distances with NAM comes of the greater rotation medial of the segments. This can be seen by comparing the posterior maxillary width, which is not affected by rotation. With both appliances, it showed similar amount of growth.

Our results are in line with other studies that investigated the maxillary growth of CLP patients with PSIO. They reported that the medial transversal width is stable with PAM, while it is reduced with NAM, and that with both appliances, the posterior transversal width shows growth [20–23]. Variations can be explained by differences in age at the second investigation period. A study from 2016 from Cerón-Zapata et al. [31] compared maxillary growth in CLP patients treated with a Hotz appliance and treated with NAM. While the study of Cerón-Zapata et al. only measured distances, our study also measured rotations of the segments. Comparing the distances measured in this study and the study of Cerón-Zapata et al. showed similar results. The distances, which show the biggest variation between the two studies, are the sagittal alveolar arch length of both segments, which show less growth in the study of Cerón-Zapata et al. However, the measurement approaches were slightly different. While our study measured the length on top of the alveolar ridge, Cerón-Zapata et al. measured on the medial side of the alveolar ridge. However, what all these studies do not show and measure are the rotation of the segments. While in previous studies the rotations of the segments were rarely measured, and if no attention was given to it, this study shows significant differences in the rotations. These differences affect directly other length in growth of the alveolar arch. This new finding needs to be taken into consideration when deciding which PSIO is the right one for the patient.

A strength of the study presented here is that the relevant points were set by four and not only one rater, as done in other studies [20, 29]. Moreover, we performed preliminary trials with all four raters by setting different anatomical and constructed points based on previous studies [20, 21, 29, 30]. In accordance to those preliminary trials, we excluded points that resulted in too much variation, and only included points that were set similarly by all four raters. Hence, it can be assumed that the “fuzziness” for the points set in this study is lower than in comparable studies. How much anatomical points variate between raters should be carefully investigated in further studies, so that there is a ground how comparable studies on the growth of the alveolar arch is.

Since this is a retrospective study, one limitation is that patients were not randomized to one of the patient groups. In addition, one treatment centre (Erlangen) only performed PAM, while the other treatment centre (Munich) only performed NAM, which might have caused a centre-related bias. Both centres, however, have a long experience in their treatment method. Another limitation of this study is the relatively low number of patients, which is due to the fact that complete non-syndromic uCLP represent only one subtype of clefts in a wide and very variable spectrum of cleft anomalies. This also leads to the fact that outliners can influence the results significantly. A higher case number would be only possible with an extension of the study period or additional cleft centres, which would lead to new sources of error and greater variability. A further limitation is the lack of a control group, which would not have received any PSIO treatment. Thereby, we could distinguish which change was caused by PSIO treatment and which was intrinsic. This is especially relevant for distances that showed growth. Some studies from the Netherlands suggest that PSIO treatment only has a temporary effect on the maxillary arch form. However, it is not clear which PSIO treatment was used in the randomized studies [32, 33]. While in our study, we observed a significant medial rotation with NAM compared to PAM over a mean of 15 weeks, the studies in the Netherlands observed no difference between PSIO treatment and no PSIO treatment, even in the short term. Thus, not all PSIO treatments may have the same effect. A short-term difference between NAM and PAM in the alveolar gap was also seen in a study of Gibson et al. However, this difference was gone by the time of the palatoplasty [34]. Also, recent studies which compare NAM with an active PSIO show no long-term difference between the PSIO treatments [35].

A treatment decision needs to consider that the greater reduction of the cleft with NAM could have a positive effect on the surgery, since with the reduction of the cleft, a presurgical convergence of the soft tissues can be reached. This might not only facilitate surgery but also help to reduce wound tension and postoperative scarring, which is illustrated as a positive effect in general surgical studies [2, 36]. Furthermore, the benefit of nasal molding and therefore more symmetric cartilage shaping needs to be taken into account. On the other hand, in cleft patients with familial transversal maxillary narrowness and/or sagittal deficiency (class III malformation) or extreme narrowness of the maxilla or collapse of the smaller segment to the median at birth, PAM might be a better option than NAM. Greater transversal and sagittal growth can reduce lateral or anterior cross-bites on the one hand and might diminish the need for secondary orthodontic interventions in childhood and adolescence or combined orthodontic-surgical treatment strategies in adulthood to correct those malocclusions and jaw misalignments.

Further studies need to evaluate if the induced changes have a long-term effect on the alveolar arch growth and if the differences between the two PSIO treatments still exist in youths or adults. Also, it should be evaluated if certain patient subgroups benefit more from PAM, while others may benefit more from NAM. Hence, future studies should focus on early life parameters enabling a decision for one of the presented treatment strategies after birth.

The relatively short treatment time with PSIO is due to the fact that it is only used before the closure of the lip with 4–6 months of life. However, already after only a short treatment period, a significant difference can be already seen between PAM and NAM. If this difference still remains after several years need to be evaluated in a follow-up study.

## Conclusion

Our study showed that both PSIO techniques affect growth of the maxilla differently until lip closure. It is unclear whether these differences translate into different long-term outcomes. Nevertheless, the short-term influence on maxillary growth induced by PSIO should be taken into account. However, follow-up studies should investigate which patients benefit the most from which PSIO technique in the long-term and focus on early life parameters allowing informed individual treatment decisions after birth.

## Supplementary Information

Below is the link to the electronic supplementary material.

Supplementary file1 (DOCX 24.1 KB)

## Data Availability

All relevant data is enclosed in the tables.

## Abbreviations

CLP: Cleft lip palate
uCLP: Unilateral cleft lip palate
NAM: Nasoalveolar molding
PAM: Passive alveolar molding
PSIO: Presurgical infant orthopaedic therapies
