# Supplementary material for: The impact of passive alveolar molding vs. nasoalveolar molding on cleft width and other parameters of maxillary growth in unilateral cleft lip palate
Source: Clin Oral Investig. 2023 Jun 23;27(9):5001–9. doi: 10.1007/s00784-023-05119-7 (PMC10492684; doi:10.1007/s00784-023-05119-7)
Supplement: Supplementary file 1 — Supplementary file1 (DOCX 24.1 KB) [file 784_2023_5119_MOESM1_ESM.docx]

Table 1 Patients characteristics PAM

| # | Age at T0 (d) | Age at T1 (d) | Gender | Cleftside | LAHSHAL-Code | Time of Treatment (d) |
| --- | --- | --- | --- | --- | --- | --- |
| 01 | 10 | 73 | m | right | LAHS--- | 63 |
| 02 | 0 | 131 | f | left | ---SHAL | 131 |
| 03 | 5 | 94 | m | left | ---SHAL | 89 |
| 04 | 1 | 139 | m | left | ---SHAL | 138 |
| 05 | 3 | 105 | m | right | LAHS--- | 102 |
| 06 | 2 | 76 | m | right | LAHS--- | 74 |
| 07 | 5 | 112 | m | left | ---SHAL | 107 |
| 08 | 2 | 125 | f | left | ---SHAL | 123 |
| 09 | 3 | 107 | f | right | LAHS--- | 104 |
| 10 | 1 | 78 | f | left | ---SHAL | 77 |
| 11 | 6 | 123 | f | right | LAHS--- | 117 |
| 12 | 3 | 94 | f | left | ---SHAL | 91 |
| 13 | 3 | 116 | m | right | LAHS--- | 113 |
| 14 | 2 | 94 | m | right | LAHS--- | 92 |
| 15 | 5 | 122 | f | right | LAHS--- | 117 |
| 16 | 6 | 118 | m | left | ---SHAL | 112 |

Note: Age at T0: the age at the time of the first plaster model in days; Age at T1: the age at the time of the second plaster model in days; Gender: m=male, f=female; LAHSHAL-Code: type of cleft: LAHS---:right cleft involving L: lip, A: alveolus, H: hard palate, S: soft palate; ---SHAL: left cleft involving S: soft palate, H: hard palate, A: alveolus, L: lip; Time of treatment: treatment duration in days

Table 2 Patients characteristics NAM

| # | Age at T0 (d) | Age at T1 (d) | Gender | Cleftside | LAHSHAL-Code | Time of Treatment (d) |
| --- | --- | --- | --- | --- | --- | --- |
| 01 | 0 | 93 | m | left | ---SHAL | 93 |
| 02 | 0 | 101 | m | left | ---SHAL | 101 |
| 03 | 1 | 92 | m | left | ---SHAL | 91 |
| 04 | 8 | 180 | m | right | LAHS--- | 172 |
| 05 | 0 | 96 | m | left | ---SHAL | 96 |
| 06 | 0 | 100 | m | left | ---SHAL | 100 |
| 07 | 1 | 73 | m | left | ---SHAL | 72 |
| 08 | 0 | 90 | m | left | ---SHAL | 90 |
| 09 | 3 | 113 | m | right | LAHS--- | 110 |
| 10 | 2 | 111 | f | right | LAHS--- | 109 |
| 11 | 0 | 95 | m | left | ---SHAL | 95 |
| 12 | 1 | 111 | m | left | ---SHAL | 110 |
| 13 | 4 | 81 | m | left | ---SHAL | 77 |

Note: Age at T0: the age at the time of the first plaster model in days; Age at T1: the age at the time of the second plaster model in days; Gender: m=male, f=female; LAHSHAL-Code: type of cleft: LAHS---:right cleft involving L: lip, A: alveolus, H: hard palate, S: soft palate; ---SHAL: left cleft involving S: soft palate, H: hard palate, A: alveolus, L: lip; Time of treatment: treatment duration in days

Table 3: Comparison sex and distances at T0 between PAM and NAM

|  | PAM | NAM | p-value |
| --- | --- | --- | --- |
| ^1^Sex m:f | 9:7 | 12:1 | 0.0443 |
| Distances/Angles at T0 |  |  |  |
| ^2^Anterior Cleft (mm) | 10.43±2.47 | 8.91±5.15 | 0.3419 |
| ^2^Anterior Maxillary Width (mm) | 25.79±2.64 | 25.15±4.20 | 0.6240 |
| ^2^Medial Maxillary Width (mm) | 31.90±2.59 | 31.80±4.19 | 0.9374 |
| ^2^Posterior Maxillary Width (mm) | 32.29±2.50 | 33.14±2.90 | 0.4039 |
| ^2^Alveolar Arch Length Great Segment (mm) | 38.57±3.12 | 36.80±3.23 | 0.1457 |
| ^2^Alveolar Arch Length Small Segment (mm) | 25.39±2.70 | 25.11±3.22 | 0.8002 |
| ^2^Sagittal maxillary length (mm) | 23.38±2.56 | 21.79±2.07 | 0.0818 |
| ^2^Lateral angle of the Great Segment (°) | 86.45±4.25 | 83.56±6.63 | 0.1661 |
| ^2^Lateral angle of the Small Segment (°) | 73.10±5.63 | 71.06±7.31 | 0.4030 |
| ^2^Medial angle of the Great Segment (°) | 89.11±5.14 | 90.14±11.05 | 0.7600 |
| ^2^Medial angle of the Small Segment (°) | 67.01±5.88 | 68.22±6.86 | 0.6135 |

^1^ The statistical test used is a Fisher exact test

^2^ The statistical test used is a t-test

Note: Values given are numbers for sex, and mean and standard deviation for other values
